# Evaluation of Rhodamine B Photocatalytic Degradation over BaTiO_3_-MnO_2_ Ceramic Materials

**DOI:** 10.3390/ma14123152

**Published:** 2021-06-08

**Authors:** Iwona Kuźniarska-Biernacka, Barbara Garbarz-Glos, Elżbieta Skiba, Waldemar Maniukiewicz, Wojciech Bąk, Maija Antonova, Susana L. H. Rebelo, Cristina Freire

**Affiliations:** 1 REQUIMTE/LAQV, Departamento de Química e Bioquímica, Faculdade de Ciências, Universidade do Porto, Rua do Campo Alegre s/n, 4169-007 Porto, Portugal; susana.rebelo@fc.up.pt (S.L.H.R.); acfreire@fc.up.pt (C.F.); 2 Institute of Technology, Pedagogical University, Podchorążych 2, 30-084 Kraków, Poland; wojciech.bak@up.krakow.pl; 3 Institute of Technology, The Jan Grodek State University in Sanok, 6 Reymonta Str., 38-500 Sanok, Poland; 4 Institute of General and Ecological Chemistry, Lodz University of Technology, Żeromskiego 116, 90-924 Łódź, Poland; elzbieta.skiba@p.lodz.pl (E.S.); waldemar.maniukiewicz@p.lodz.pl (W.M.); 5 Institute of Solid State Physics, University of Latvia, Kengaraga 8, LV-1063 Riga, Latvia; Maija.Antonova@cfi.lu.lv

**Keywords:** perovskite, photo-oxidation, water treatment, photocatalysts, impedance spectroscopy

## Abstract

Ferroelectric ceramics (BaTiO_3__MnO_2_) with different Mn admixtures were prepared using solid-state synthesis. Elemental analysis, powder X-ray diffraction, scanning electron microscopy, Fourier-transform infrared spectroscopy, and impedance spectroscopy confirmed that the BaTiO_3_ and MnO_2_ coexisted in the ceramics. In addition, the high purity and homogeneity of the element distributions in the ceramic samples were confirmed. The adsorptive and photocatalytic properties of the BaTiO_3_ (reference sample, BTO) and BaTiO_3__MnO_2_ materials (BTO_x, where x is wt.% of MnO_2_ and x = 1, 2 or 3, denoted as BTO_1, BTO_2 and BTO_3, respectively) were evaluated using Rhodamine B (RhB) as the model dye in a photocatalytic chamber equipped with a UV lamp (15 W) in the absence of additional oxidants and (co)catalysts. No adsorption of RhB dye was found for all the materials during 360 min (dark experiment). All samples were photocatalytically active, and the best results were observed for the BTO_3 material, where RhB was 70% removed from aqueous solution during 360 min of irradiation. The photodegradation of RhB in the presence of MnO_2_-modified BTO ceramics followed a pseudo-first order model and the rate constant of BTO_3 was about 10 times higher than that of BTO, 2 times that of BTO_2, and 1.5 times that of BTO_1. The photocatalysts could be successfully reused after thermal activation.

## 1. Introduction

Semiconductor photocatalysts, which hold great potential for converting solar energy to chemical energy, have been proven to be available and promising materials for environmental remediation [1,2,3]. The key to enhancing the photocatalytic activity of semiconductor mainly lies in the effective combination of photon absorption, bulk diffusion and the separation of a photoinduced charge [4,5]. Unfortunately, most semiconductor oxides generally have a wide band gap and a relatively high recombination rate of electron–hole pairs, leading to poor efficiency of the photocatalytic reaction.

Since the spontaneous polarization of ferroelectrics is advantageous for the separation of photogenerated charge carriers, many efforts have been devoted to promoting the photocatalytic performance of ferroelectric materials [6]. Nevertheless, few studies on light absorption improvements of traditional ferroelectrics have been reported so far.

Perovskite oxides, with the general formula ABO_3_, are the most propitious photocatalysts studied in the field. Here the terms A and B refer to different metallic cations. Predominantly, the ‘A’ cation is either an alkali, alkaline, or rare earth metal cation, whereas the ‘B’ cation is regularly a transition metal element, with a size smaller than the A cation [2]. Especially, barium titanate with a perovskite structure has been attracting extensive attention due to its excellent dielectric properties in lead-free devices, such as multilayer capacitors, thermistors, electro-optical devices and electromechanical devices [7,8]. However, to our knowledge, few reports describe the promising photocatalytic properties of BaTiO_3_, probably due to its large band gap [5]. Furthermore, the intrinsic performance of the photocatalyst can be reduced by a high rate of charge recombination. 

To match band potentials, many efforts have been made by constructing a heterojunction interface to enhance the charge separation between different semiconductors. Recently, several transition metal oxides, such as Co_3_O_4_ [9,10] or CoO_x_ [11,12], MnO_x_ [13,14], FeO_x_ [15,16], CuO_x_ [13,17], and NiO [13,18], have been developed as low-cost and efficient (co)catalysts for photocatalytic reactions. Generally, metal oxides can act as reduction (co)catalysts and oxidation (co)catalysts, which trap photogenerated electrons and holes, respectively, thus providing active sites for photocatalytic redox reactions and can extend the absorption range from UV to the visible light region [18]. Oxidation (co)catalysts, including CoO_x_, MnO_x_, Fe_2_O_3_, RuO_2_, and IrO_x_, are used to capture holes and act as active sites for oxidation reactions and enhance the photocatalytic activity of the system. To the best of our knowledge, there are few reports on (co)catalyst-modified ferroelectric BaTiO_3_ and its applications in water remediation. 

In this paper, BaTiO_3_-MnO_2_ ceramics were prepared using one-step thermal synthesis and their photocatalytic activities were assessed through oxidative photodegradation of a typical organic dye, Rhodamine B. The amount effect of (co)catalysts on the photocatalytic activity, photodegradation pathway, and mechanisms were also systematically discussed.

## 2. Materials and Methods

### 2.1. Materials, Reagents and Solvents

All reagents used during the experimental work were used as received without further purification: barium carbonate (99.98%), titanium oxide (≥99.9%), manganese (IV) oxide (≥99%), potassium bromide (≥99%, FTIR grade), hydrogen peroxide 30 wt.% in H_2_O (ACS reagent), rhodamine B (≥98% HPLC grade, RhB) and Nujol mull were purchased from Sigma-Aldrich, Darmstadt, Germany. Ethanol (99.8% pure p.a.,) was obtained from Chempur, Karlsruhe, Germany. Ultrapure water, (Millipore, Interlab, Wellington, New Zealand, specific resistivity 18 MΩ cm) was used throughout the experiments.

Preparation of barium titanate ceramics.

The preparation procedure of polycrystalline samples of BaTiO_3_ and BaTiO_3_ modified with different MnO_2_ contents, denoted as BTO and BTO_1, BTO_2, and BTO_3, respectively, for 1, 2, and 3 wt.% of MnO_2_, were published elsewhere [7]. Briefly, the samples were synthesized from an analytically pure barium carbonate (BaCO_3_), titanium oxide (TiO_2_), and manganese oxide (MnO_2_) depending on the desired material composition. A mixture of the raw materials was homogenized and ground in an agate ball mill in ethanol for 24 h. The dried material was calcined at a temperature of 1523 K for 1–2 h. After calcination, the powder was ground in ethanol, cold-pressed (100 MPa), and then sintered for 2–3 h with the aid of conventional ceramic technology in the temperature range of 1633–1693 K, depending on the composition. 

### 2.2. Chemical and Physicochemical Characterization

The content of Mn in the ceramic material samples was determined by high-resolution continuum source atomic absorption spectrometry (HR-CS AAS) using a ContrAA 300 atomic absorption spectrometer (Analytik Jena, Jena, Germany) operating in the flame (air–acetylene) mode. A total of 50 mg of the ceramics containing Mn were digested using the Anton Paar Multiwave 3000, Graz, Austria, closed system instrument. Prior to proper analyses, the two-step procedure of sample preparation was applied. The initial digestion of the ceramic samples with the mixture of concentrated acids (HCl, HF and HNO_3_ (2:1:1, *v*/*v*)) was followed by the complexation with saturated H_3_BO_3_ solution, which supports free fluoride ion complexation and facilitates the dissolution of precipitated fluorides.

The microstructure of the surface of our polycrystalline samples was examined using a SEM Model Hitachi S4700, Tokyo, Japan with a field emission and a Noran Vantage Waltham, MA, USA, EDS system. Energy-dispersive X-ray spectroscopy (X-ray EDS) microanalysis was applied to investigate the homogeneity of composition and an electron probe microbeam analysis (EPMA) was used to analyze the distribution of elements at sample surfaces. Investigations of the chemical compositions were performed using a Noran-Vantage Waltham, MA, USA microanalyzer, which was a part of the Hitachi SEM. The use of a lithium-drifted siliceous detector with a multi-channel pulse height analyzer enabled us to obtain diffraction patterns from any chosen micro-regions of the sample surfaces. 

FTIR spectra were obtained using KBr pellets (Merck Darmstadt, Germany, spectroscopic grade), containing 0.4 wt.% material, 1:250 sample KBr ratio using a Jasco FT-IR 460 Plus spectrometer, Pfungstadt, Germany. All spectra were collected at room temperature, in the 400−4000 cm^−1^ range, using a resolution of 4 cm^−1^ and 32 scans. 

X-ray powder diffraction (XRD) patterns were collected using a PANalytical X’Pert Pro MPD diffractometer in the Bragg–Brentano reflection geometry with CuKα radiation from a sealed tube (Malvern Panalytical Ltd., Royston, UK). The apparatus operates in the range of 2θ = 5–90° with a step size of 0.0167°.

The impedance was measured using a Novocontrol Alpha High-Resolution Dielectric Analyzer Montabaur, Germany in the temperature range of 173 K to 523 K and at a frequency varying from 0.1 Hz to 10 MHz. Silver paint was used on the polished surfaces as electrodes. Nitrogen gas was used as a heating and cooling agent. 

Ultraviolet–visible (UV–Vis) spectra for solutions and solid samples were registered on a Varian Cary50Bio spectrophotometer, CA, USA, in the range of 650–200 nm using a quartz cell with path lengths of l = 1 cm. The electronic spectra of the solids were measured using Nujol mull. 

### 2.3. Adsorption and Photocatalytic Performance Tests

The adsorption properties and the photocatalytic activity of the obtained BTO and MnO_2_-modified BTO ceramics were evaluated using RhB as a model compound. The adsorption experiments were carried out by agitating 100 mg of catalyst with 100 mL of aqueous RhB solution (4.8 mg L^−1^) at 200 rpm, for 360 min in the dark. The samples were collected at fixed time intervals and analyzed using UV–Vis spectrometry. The photocatalytic activity of the obtained ceramics was evaluated, after the adsorption studies, under similar experimental conditions to those applied in the adsorption tests, but under light irradiation. As a light source, a UVA lamp (15 Watt) (Osram, Berlin, Germany) was used. The general procedure was carried out as follows: 100 mL of aqueous RhB solution (4.8 mg L^−1^) was placed in a water-jacketed reactor. Then, 100 mg of catalyst was suspended in the solution. The suspension was air bubbled for 360 min in the dark to establish adsorption–desorption equilibrium of RhB. The suspension was then irradiated under UV. Samples were withdrawn periodically from the reactor, centrifuged, and then analyzed by recording the absorption spectra of RhB. The concentration of RhB dye was calculated using a standard curve (A = 0.2058c–0.0013 (R^2^ = 0.999) where A is absorbance and c is the RhB concentration in mg L^−1^). The maximum absorption of RhB in water was at a wavelength of 554 nm. The reproducibility of the results was checked by repeating the photocatalytic tests at least two times and it was found to be within acceptable limits (<1%). Blank experiments were performed in the absence of the photocatalysts under light irradiation.

## 3. Results

### 3.1. Characterisation of the Materials

The manganese concentrations in BTO_1, BTO_2, and BTO_3 materials were analyzed via HR-AAS. The contents of Mn expressed as MnO_2_ wt% incorporated into the ceramic, before and after catalytic tests, are compared in Table 1. Chemical analyses confirmed the predicted Mn concentration in each of the obtained materials. Furthermore, the results were confirmed by EDS (Table 1), which revealed that the stoichiometry of all samples was maintained (according to assumptions). A good correlation between manganese determination in synthesized materials using the bulk solution method and using the EDS method was observed for each sample.

**Figure 1:**
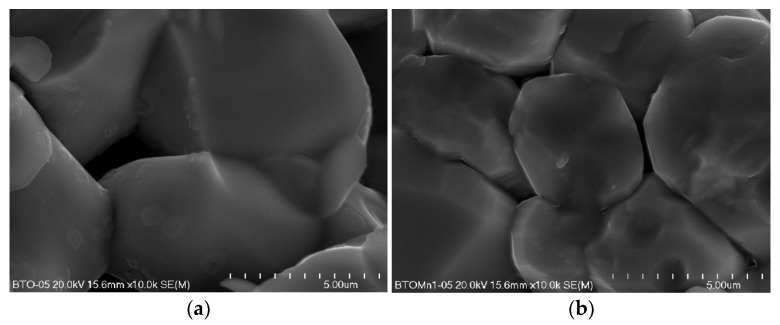

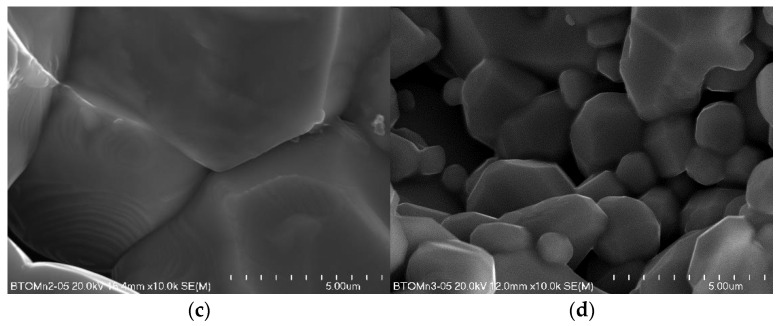
SEM images of ceramic BaTiO_3_ (**a**), BTO_1 (**b**), BTO_2 (**c**), and BTO_3 (**d**) (magnification 10,000×).

SEM images for BTO and BTO-doped MnO_2_ samples are presented in Figure 1. In all investigated materials, a well-developed microstructure with integrated growth terraces was observed, indicating that the growth of grains took place according to the layer mechanism. The SEM image of pure BTO (Figure 1a) shows that its microstructure consists of inter-granular pores and grains of various sizes (average grain size is estimated to be about 10 μm). The micrographs of the samples with MnO_2_ (BTO_1, BTO_2, and BTO_3, Figure 1b–d) indicate that the MnO_2_ admixture influenced the microstructure of the materials. For all samples where MnO_2_ was used as an admixture, an inhibiting effect on the grain growth process was observed. Consequently, a relatively homogeneous microstructure arose, with grains smaller than those typical for the non-modified sample (BTO). The average grain size (estimated using the intercept line method) for the materials was about 6 μm, 8 μm, and 3 μm respectively for BTO_1, BTO_2, and BTO_3. This may be a consequence of the experimental conditions of the sintering process (thermal treatment) and the behavior of Mn^4+^-ions in the BTO ceramics.

Obtained elemental distribution mappings (measured using EPMA) confirmed the presumed qualitative composition for the samples under examination (Figure S1, Supplementary Materials). Additionally, the homogenous distribution of Mn on the surface and interior of perovskite samples was confirmed using the HR-Cs AAS and EDS methods.

**Figure 2:**
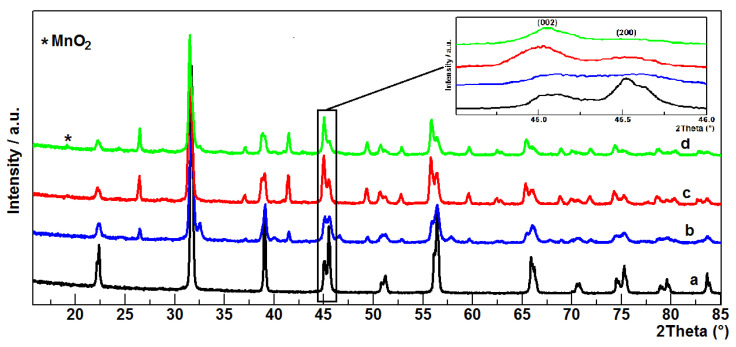
XRD patterns of BTO (**a**), BTO_1 (**b**), BTO_2 (**c**), and BTO_3 (**d**). Insert: enlargement of XRD patterns in the 2θ range of 44.5–46.0°.

The crystalline structures of the obtained ceramics were analyzed using powder XRD. Figure 2 shows the X-ray diffraction patterns of BTO, BTO_1, BTO_2, and BTO_3. The XRD results indicate that, for the BTO sample, only tetragonal BaTiO_3_ structures with the P4mm space group (JCPDS data No. 05-0626), while, for the other samples, we can see a mixture of two BaTiO_3_ polymorphs. This is evidenced, for example, by the appearance of additional peaks in the diffraction patterns at 2θ for angles of 24.45° and 41.44°. Similarly, as in the BTO sample, the major crystalline phase in BTO_1, BTO_2, and BTO_3 showed good agreement with the tetragonal BaTiO_3_ structure. In all diffraction patterns we observe a peak splitting at 2θ of about 45° (see Figure 2 insert), which indicates a significant degree of tetragonality [19]. In general, the XRD patterns of the tetragonal BaTiO_3_ show split peaks at 45°, corresponding to Miller (hkl) indexes (002) and (200), whereas cubicBaTiO_3_ (JCPDS data No. 31-0174) has only one peak at 45°, corresponding to the (002) plane. The minor crystalline phase in BTO_1, BTO_2, and BTO_3 represents a hexagonal BaTiO_3_ structure with the P63/mmc space group (JCPDS data No. 034-0129). The appearance of two BaTiO_3_ polymorphs may be due to the presence of MnO_2_ in the system. In addition, for the BTO_2 and BTO_3 samples with MnO_2_, a low intensity peak appears at 2θ, ca. 19.1°, in both diffractograms. This peak shows good agreement with the cubic MnO_2_ (111) plane (JCPDS data No. 042-1169). The absence of other major peaks, e.g., (311) or (400) at 2θ of 37.06° and 45.09°, respectively, may indicate a good MnO_2_ dispersion in BaTiO_3_.

**Figure 3:**
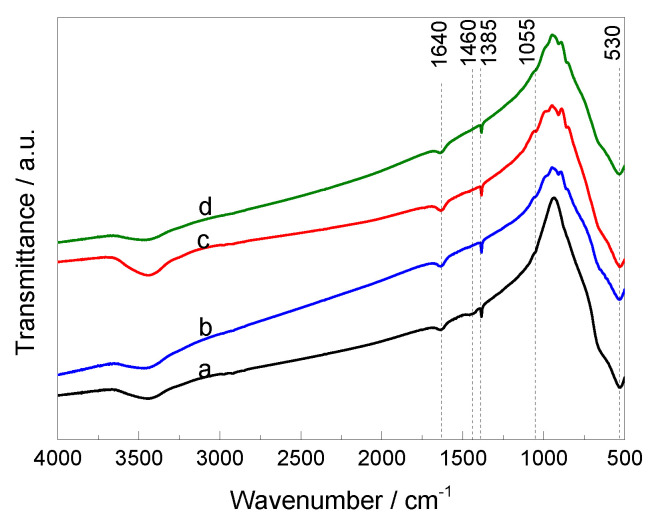
FTIR spectra of BaTiO_3_ ceramics: BTO (**a**), BTO_1 (**b**), BTO_2 (**c**), and BTO_3 (**d**) in the 500–4000 cm^−1^ range.

The ceramics were also characterized by FTIR spectroscopy in order to gain further insight into their chemical structures. Figure 3 shows the FTIR spectra of BTO and BTO modified with MnO_2_ materials. For all ceramics, the broad low-intensity band with a maximum at 3495 cm^−1^ was assigned to the O−H stretching vibrations of weakly bound water interacting with its environment via hydrogen bonding [20], and at 1640 cm^−1^ usually assigned to the bending H−O−H vibration of water absorbed on the materials [21,22]. The low intensity bands at 1460, 1385, and 1055 cm^−1^ are characteristic for CO_3_^2−^ anion vibrations from traces of BaCO_3_. These bands are associated, respectively, with the asymmetric stretching vibrations, the symmetric stretching vibrations, and the bending out of plane vibrations in the CO_3_^2−^ group [23,24]. The presence of small amounts of BaCO_3_ in the BTO ceramics may be caused by the excess BaCO_3_ used in the preparation of the materials and due to it not completely decomposing under the preparation conditions, as was observed for BaTiO_3_ nanopowders prepared via wet routes [25].

All the ceramics show a strong band at about 530 cm^−1^, usually assigned to the Ti−O stretching mode [26]. On the other hand, most of the vibration peaks related to the presence of MnO_2_ in the sample should appear at 200–450, 450–600, and 600–750 cm^−1^ [21,27,28]. The bands expected at 450–600 and 600–750 cm^−1^ are overlapped by a strong band at 530 cm^−1^ (Ti−O) and the others expected at 200–450 cm^−1^ are out of the measurement range. New weak bands in the region of 800–1000 cm^−1^ were observed for BTO doped with MnO_2_ in comparison to bare BTO. These bands were also observed for α-MnO_2_ and Ag@MnO_2_ [27,29], which confirmed the presence of MnO_2_ in BTO-modified samples. The results are consistent with those obtained from other characterization methods (HR-CS AAS, SEM-EDS, and XRD) and confirmed that BaTiO_3_ and MnO_2_ coexist in the BTO-based ceramics.

The electronic spectra of the BTO and BTO modified with MnO_2_ ceramics (solid samples) in Nujol mull are given in Figure S2 of the Supplementary Materials. The ceramics exhibit absorption mainly in the UV region; the presence of MnO_2_ in the ceramics does not significantly increase the absorption pattern in the low energy region due to the low concentration of MnO_2_ in the samples. The band gap energies were calculated according to the equation E_g_ = hc/λ, where E_g_ is the band gap energy (eV), h is Planck’s constant (4.135667 × 10^−15^ eV s), c is the velocity of light (3 × 10^8^ m/s), and λ is the wavelength (nm) of the absorption onset. The band gap energies were 4.5, 4.1, 3.7, and 3.5 eV for BTO, BTO_1, BTO_2, and BTO_3, respectively. Thus, it could be inferred that the band gap of BTO became narrower through the addition of MnO_2_, but that the photocatalyst should still be active under UV irradiation.

### 3.2. Adsorption Experiments

Preliminary control adsorption experiments were performed under dark conditions. The RhB removal efficiency of BTO and BTO-based ceramics was less than 5% during 360 min of contact. Due to this, the sorption kinetics were not studied further for any of these systems as the adsorption process did not show a significant effect on RhB removal.

### 3.3. Photocatalytic Tests

**Figure 4:**
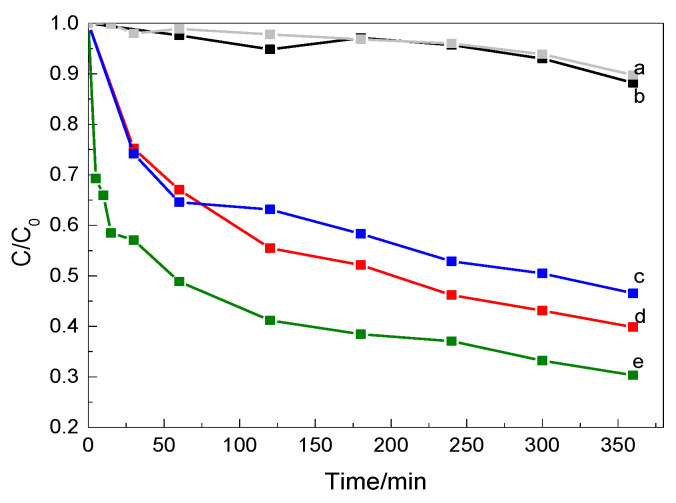
Photocatalytic degradation of RhB by different photocatalysts: none (**a**), BTO (**b**), BTO_1 (**c**), BTO_2 (**d**), and BTO_3 (**e**).

To demonstrate the effect of ferroelectric polarization on the photocatalytic activity of MnO_2_-modified BTO ceramics, their activity in the degradation of RhB (organic pollutant model) was evaluated under light irradiation without the addition of any oxidants. For comparison, the photodegradation ability of pure BaTiO_3_ ceramic was also evaluated under the same experimental conditions. The results, including a blank experiment (without photocatalysts), are summarized in Figure 4. All the MnO_2_-modified BTO ceramics exhibited enhanced photocatalytic activity in comparison with the pure BTO. BTO_3 ceramic showed the best photodegradation activity, which was 5.8 times greater than that of pure BTO ceramic, after 360 min of photocatalysis.

Some RhB degradation under light irradiation was observed in the absence of catalyst (ca. 10% after 360 min of irradiation), indicating that a charge transfer process between the dye and catalyst may be an important factor for improving dye degradation [30]. The pure BTO, during 360 min of irradiation, led to a 12% degradation of RhB. This activity is nearly the same to that observed in the absence of catalysts; therefore, it can be assumed that BTO does not show photocatalytic activity in RhB degradation. The results showed that BTO modification with MnO_2_ is necessary to induce an efficient photocatalytic activity. Moreover, the weight ratio of BTO to MnO_2_ in the ceramics also played an important role in the photocatalytic activity. The degradation of RhB increased in following order: BTO_1 (53%) < BTO_2 (60%) < BTO_3 (70%). 

To confirm that the storage conditions did not affect the active phase of the photocatalysts, catalytic tests were performed 24 h after thermal activation at 573 K. The results showed comparable activity in RhB photodegradation (1st cycle) for samples stored for one year (in air at room temperature) and freshly activated samples. This suggests that the storage conditions did not affect the active phase of these photocatalysts, over at least one year of storage.

The photocatalytic degradation of RhB, similar to the photocatalytic decolorization of most organic compounds, follows the Langmuir−Hinshelwood model [31]. The model was developed by Turchi and Ollis [32] and is expressed as:

(1) $$r_{i}=-\frac{dC_{i}}{dt}=\frac{kKC_{i}}{1+kKC_{i}}$$

where *C_i_* is the molar concentration of the dye solution, *k* is the reaction rate constant, and K is the adsorption coefficient of the dye to the catalysts. When *C_i_* is small (*C_i_* << 10^−3^ M) *kKCi* << 1, (1) will be simplified to a pseudo-first-order equation:

(2) $$r_{i}=-\frac{dC_{i}}{dt}=kKC_{i}$$

Integrating Equation (2) gives the following relationship:

(3) $$\ln\left(\frac{C}{C_{0}}\right)=-kt$$

where *C*_0_ and *C* (mg L^−1^) are the concentrations of RhB at *t* = 0 and time *t* (min), respectively, and *k* (min^−1^) is the rate constant. 

**Figure 5:**
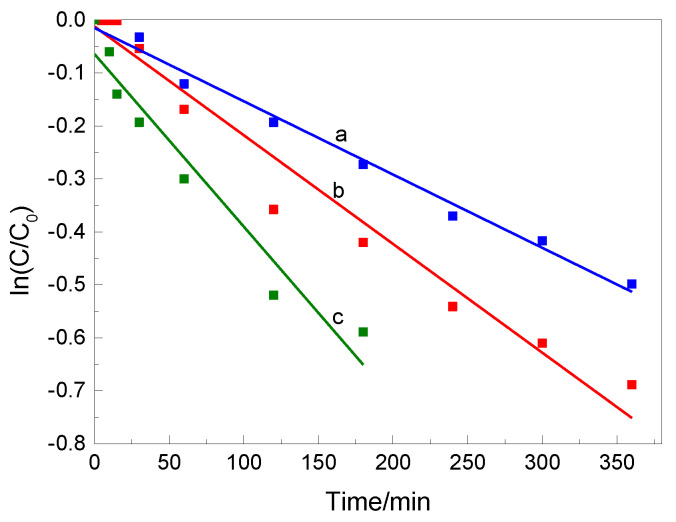
First-order kinetic fitting curves of RhB photodegradation under UV light irradiation using different photocatalysts: BTO_1 (**a**), BTO_2 (**b**), and BTO_3 (**c**).

The data fitting of RhB photocatalytic degradation in the presence of BTO modified with MnO_2_ samples under UV irradiation is shown in Figure 5; the respective equations, fitted rate constant values, and correlation coefficients are collected in Table S1 in the Supplementary Material.

As shown in Figure 4 and Figure 5, the reaction rate of BTO_3 (k = 3.3 × 10^−3^ min^−1^) was about 10 times that of BTO (k = 3.0 × 10^−4^ min^−1^), 2 times that of BTO_1 (k = 1.4 × 10^−3^ min^−1^), and 1.5 times that of BTO_2 (k = 1.9 × 10^−3^ min^−1^), respectively. Obviously, BTO_3 presented a much higher photocatalytic efficiency than pure BTO or BTO with (co)catalysts loaded with lower MnO_2_ quantities. This might be due to the fact that MnO_2_ promotes the migration of holes and, therefore, more charge carriers could participate in the reaction. The changes in the absorption spectra of RhB dye solutions during irradiation showed a shift towards lower wavelengths of the absorption maximum of the peak at 554 nm when all the BTO modified with MnO_2_ photocatalysts were employed. This hypsochromic shift in the maximum absorption peak (λ_max_ = 554 nm) of RhB solution during photocatalysis has been widely reported in the literature [33,34]. It has been explained as being the result a series of N-deethylated intermediates of RhB, i.e., the four ethyl groups of the RhB molecule are removed in a stepwise manner. In addition to deethylation, one other process that occurs during photocatalytic degradation of RhB is the cleavage of the conjugated chromophore structure. These two processes coexist and compete with each other [33].

**Figure 6:**
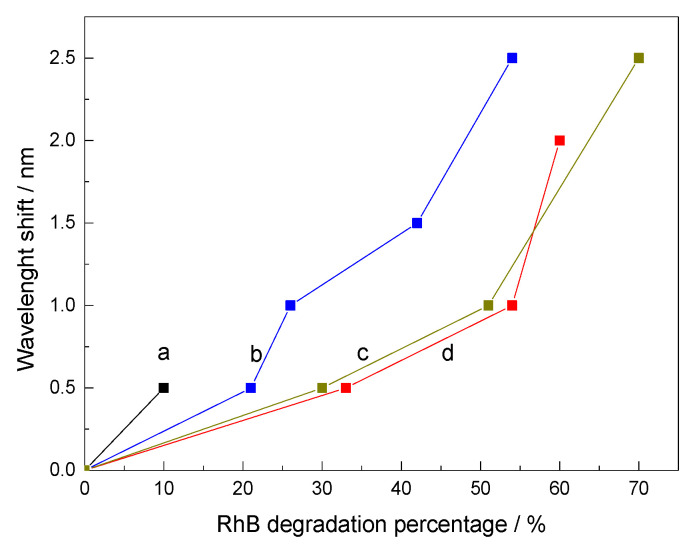
The maximum absorption wavelength shift (λ_max_ = 554 nm) of RhB as a function of degradation percentage in the presence of BTO (**a**), BTO_2 (**b**), BTO_3 (**c**), and BTO_1 (**d**) photocatalysts during 360 min of irradiation.

The decrease in the maximum of adsorption in the presence of BTO_1, BTO_2, and BTO_3 photocatalysts suggests that RhB suffers a rather facile cleavage of the whole conjugated chromophore (Figures S3–S6, Supplementary Materials). For all MnO_2_-containing photocatalysts, in the early stage of light irradiation, the absorption band shift can be ignored because of the high concentration of RhB and the poor yield of N-deethylated intermediates. However, for further RhB degradation, and in the stepwise appearance of intermediates at a later stage, the spectral blue shift becomes more pronounced. As observed in Figure 6, the vague hypsochromic shift in all BTO-MnO_2_ systems indicated that the chromophore cleavage predominates over deethylation. A similar effect was observed for BTO-MnO_x_ obtained using a photodeposition method [33].

**Figure 7:**
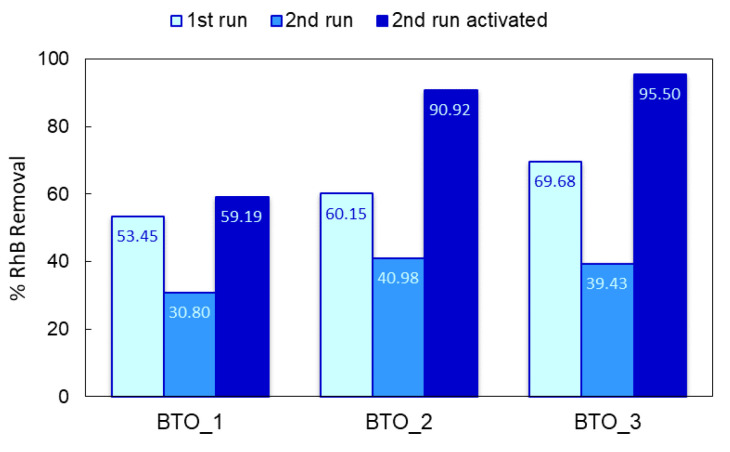
Comparison of the RhB removal between the original (1st run), reused without thermal regeneration (2nd run), and thermally regenerated catalysts (2nd run activated). Time = 360 min.

To evaluate the stability of the BTO-MnO_2_ ceramic catalysts, a set of recycling and reuse experiments were performed for the degradation of RhB contaminant under light irradiation, as shown in Figure 7. After the first catalytic run, the catalysts were washed with water and ethanol and dried in air at 323 K. During the second photocatalytic cycle, the catalysts presented a much lower contaminant removal ratio than in the first one. This significant decrease in photocatalytic activity of the second reaction cycle might be due to deactivation of active sites of the catalysts in the first reaction cycle or from the adsorption of partially degraded products of RhB acting as blocking agents, or from changes in the BTO structure during light irradiation. The adsorption studies (dark experiments) suggest that the former case does not occur, as the RhB adsorption efficiency is less than 5%. Thus, the lowering of the catalysts activity in the second cycle may be due to changes in the ceramics. Indeed, when the photocatalysts were dried at 573 K between catalytic cycles, the catalysts presented a similar or better contaminant removal than that observed in the first cycle, Figure 7. This suggests that light irradiation leads to defects in the perovskite structure. The presence of these defects decreases the electrical resistance of the samples and may lead to better photoactivity of the catalysts. This indicated that the BTO-based ceramics are stable under low-power UV irradiation (15 W), which is important for practical applications. 

**Figure 8:**
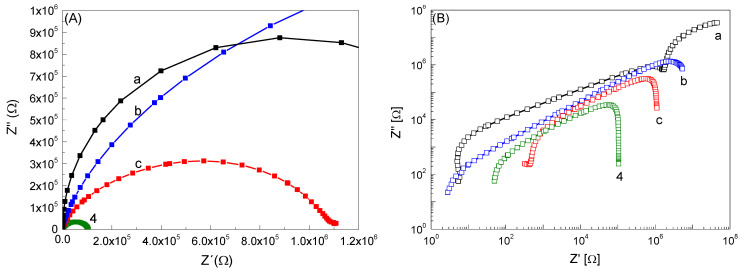
Complex impedance plot of Z’ vs Z’ at 473 K for BTO (**a**), BTO_1 (**b**), BTO_2 (**c**), BTO_3 (**d**) ceramics as Nyquist plots in linear (**A**) and log–log (**B**) systems.

The electrical behavior of the samples was studied over a wide range of temperatures and frequencies using impedance spectroscopy (IS). The typical complex impedance plots of all BaTiO_3_ modified with MnO_2_ ceramics at 473 K are shown in Figure 8. The impedance data are presented as Nyquist plots in linear (Figure 8A) and log–log (Figure 8B) systems. The impedance spectrum is only a segment of an arc due to the limit of measuring frequency. Usually, a conduction process results in a semi-circular arc in a linear complex plane plot. The log–log presentation in Figure 8B enables us to compare the measured impedance of all the samples in one plot. Moreover, the logarithmic plot still offers significant advantages in several respects, which were discussed by Jonscher [35]. The plot for the BaTiO_3_ ceramic in Figure 8B confirms the existence of two semi-circle arcs that are not visible in Figure 8A. In the impedance spectra, we attribute the higher frequency response (small arc) to the grains, and the lower one to the grain boundaries [36]. Other samples with additions of MnO_2_ show one semi-circle arc. The dimensions of the semi-circular arcs correspond to the resistance values of the measured ceramics. In Figure 8B, we can see a decrease in the impedance with an increase in wt.% of MnO_2_. Furthermore, all semi-circular arcs shift from right to left and have reduced diameters as temperature increases due to the associated decreases in impedances (not shown here). The observed decrease in the radius value of the semi-circle with increasing manganese content may be related to a faster rate of separation and transfer of electron–hole pairs, wherein a larger radius indicates a larger charge transfer resistance; thus, a lower separation efficiency of photogenerated electron–hole pairs.

Comparison with the literature data on RhB photodegradation efficiency over ferroelectric semiconductors is not easy, especially due to different preparation methods of BTO, and operating conditions of the photocatalysis (RhB concentration, photocatalyst mass ratio, and the lamp power, etc.) used by other authors. The materials prepared and studied in this work are satisfactory considering the operating parameters (lamp power (200 [37], 300 [8,33,38] or 500 W [5,39] versus 15 Watt in this work), the dosage of photocatalysts (3.0 g L^−1^ [31,38] versus 1.0 g L^−1^ in this work), etc.). This confirms the superiority of the resulting materials in the elimination of organic dyes in wastewaters. For nitrogen-doped BaTiO_3_ particles (N-BTO) synthesized by addition of urea and a sintering of solid sample, the best RhB photodegradation activity was found for N_BTO (1:20, BTO:urea mass ratio) [8]. The best photodegradation efficiency was nearly 50% after 250 min of irradiation (300 W Xe lamp), whereas, for other BTO:urea ratios, the efficiencies were much lower (less than 35%) under the same experimental conditions. The BTO-MnO_x_ obtained using the photodeposition method leads to 28.3% RhB degradation after 120 min of full spectrum irradiation [33]. These values are obviously lower than that found in this work (70% of RhB degradation for BTO_3 photocatalyst). Comparable RhB photodegradation efficiency to that obtained in this work was found for Bi_2_O_3_-BTO composite obtained by a impregnating–annealing method (70% of RhB degradation after 240 min of visible light irradiation) [5]. Slightly higher RhB photodegradation efficiency than that found in this work was found for metal oxide and BTO composites during a photo-oxidation process. For BTO-Fe_2_O_3_, the RhB photodegradation efficiency depends on the weight ratios of BTO:Fe_2_O_3_ and it was found to be nearly 100% after 120 min of UV-light irradiation for BTO-Fe_2_O_3_, with a 1:0.08 weight ratio. For the materials with higher Fe_2_O_3_ loading, the RhB degradation efficiency was much lower and dropped to 20% for BTO-Fe_2_O_3_ (1:08, BTO:Fe_2_O_3_ ratio) [38]. 

For the series of BTO-TiO_2_ core–shell heterostructures with different BTO:TiO_2_ weight ratios, the best photodegradation activity, was found for the 1.2:1 ratio (RhB photodegradation nearly 80%) and it was 1.8 times greater than that of the pure TiO_2_ nanoparticles after a 120 min of UV-light irradiation [37]. The best results were found for BaTiO_3_ annealed with nanostructured Ag. This catalyst almost completely degraded the RhB after 60 min of photocatalysis [31]. For ZnO-BaTiO_3_ heterostructures, high (97%) RhB degradation was achieved during 30 min of ultrasound (power 200 W) together with light irradiation [39].

The photocatalysts after recyclability tests were further characterized. The content of manganese in BTO_1, BTO_2, and BTO_3 materials was confirmed using the HR-CS AAS method (Table 1). The results show that the MnO_2_ contents prior to and after the catalytic test remained unchanged; thus, no leaching into the reaction media was observed. In addition, the good chemical stability of the BTO-based photocatalysts was confirmed by FTIR. There was no significant change between the FTIR spectra of as-prepared composites and those after photocatalytic tests, suggesting the stability of the materials and this confirmed that no RhB was adsorbed/retained in the material matrix during the photocatalytic process. All these results reveal that the photocatalyst has excellent stability and great potential application value.

## 4. Conclusions

In this paper, modification of ferroelectric BaTiO_3_ with MnO_2_ was successfully achieved via a one-step thermal synthesis method. To the best of our knowledge, this is the first systematic report on MnO_2_-modified ferroelectric BTO and its application in the oxidative photodegradation of an organic dye (RhB). The composition and purity of the resulting photocatalysts were confirmed using standard methods. With respect to the MnO_2_ percentage of loaded BTO, BTO_3 (3 wt.% of MnO_2_) showed a much higher photocatalytic activity in the degradation of RhB under light irradiation, and its reaction rate is 10-fold, 2-fold, and 1.5-fold that of BTO, BTO_2, and BTO_1. The degradation pathway is proved to be the same for BTO and BaTiO_3_ modified with MnO_2_ systems, and UV–Vis studies indicate that the chromophore cleavage predominates over deethylation of RhB. During catalyst reuse studies, it was shown that thermal activation was necessary as it leads to an improvement in photocatalytic activity.

## Figures and Tables

**Table 1:** Loading of MnO_2_ in perovskite ceramics obtained from HR-CS AAS and EDS methods.

Sample	MnO_2_ Content (%)		
	As Prepared		After Catalytic Test
BTO_1	0.94 ^1^	0.98 ^2^	0.96 ^1^
BTO_2	2.05 ^1^	2.02 ^2^	2.13 ^1^
BTO_3	2.77 ^1^	2.82 ^2^	2.98 ^1^

^1^ Mn content in wt.% obtained by HR-CS AAS, ^2^ Mn content in wt.% obtained by SEM-EDS.

## Data Availability

The data presented in this study are available on request from the corresponding author.

## Supplementary Materials

The following are available online at https://www.mdpi.com/article/10.3390/ma14123152/s1. Table S1. Photodecolorization of RhB and calculated reaction rates of different photocatalysts; Figure S1. Elemental area-mappings for BTO, BTO_1 and BTO_3, representative examples BTO; Figure S2. UV-Vis absorption spectra of BaTiO_3_ ceramics: BTO (a), BTO_1 (b), BTO_2 (c) and BTO_3 (d) in 220-500 cm^−1^ range in Nujol mul; Figure S3. UV-Vis absorption spectra of RhB dye solution with different irradiation time using BTO_1 as photocatalyst; Figure S4. UV-Vis absorption spectra of RhB dye solution with different irradiation time using BTO_2 as photocatalyst; Figure S5. UV-Vis absorption spectra of RhB dye solution with different irradiation time using BTO_3 as photocatalyst; Figure S6. FTIR spectra of BTO_1 (a), BTO_2 (b), BTO_3 (c) and BTO (d) after second photocatalytic cycle.
