# Supplementary material for: Evaluation of Rhodamine B Photocatalytic Degradation over BaTiO3-MnO2 Ceramic Materials
Source: Materials (Basel). 2021 Jun 8;14(12):3152. doi: 10.3390/ma14123152 (PMC8228469; doi:10.3390/ma14123152)

materials

MDPI

Supplementary Materials

Evaluation of Rhodamine B Photocatalytic Degradation over $BaTiO_3$-MnO_2 Ceramic Materials

Iwona Kuźniarska-Biernacka [1,*], Barbara Garbarz-Glos [2,3,*], Elżbieta Skiba [4], Waldemar Maniukiewicz [4], Wojciech Bąk [2], Maija Antonova [5], Susana L. H. Rebelo [1] and Cristina Freire [1]

1 REQUIMTE/LAQV, Departamento de Química e Bioquímica, Faculdade de Ciências, Universidade do Porto, Rua do Campo Alegre s/n, 4169-007 Porto, Portugal; susana.rebelo@fc.up.pt (S.L.H.R.); acfreire@fc.up.pt (C.F.)
2 Institute of Technology, Pedagogical University, Podchorążych 2, 30-084 Kraków, Poland; wojciech.bak@up.krakow.pl
3 Institute of Technology, The Jan Grodek State University in Sanok, 6 Reymonta Str., 38-500 Sanok, Poland
4 Institute of General and Ecological Chemistry, Lodz University of Technology, Żeromskiego 116, 90-924 Łódź, Poland; elzbieta.skiba@p.lodz.pl (E.S.); waldemar.maniukiewicz@p.lodz.pl (W.M.)
5 Institute of Solid State Physics, University of Latvia, Kengaraga 8, LV-1063 Riga, Latvia; Maija.Antonova@cfi.lu.lv
* Correspondence: iwonakb@fc.up.pt (I.K.-B.); barbara.garbarz-glos@up.krakow.pl (B.G.-G.)

Table S1. Photodecolorization of RhB and calculated reaction rates of different photocatalysts.

Photocatalyst	C/C_0	k^a (min^{-1})	R^2
BTO	0.88	3.0×10^{-4}	0.78
BTO_1	0.47 (0.76)^b	1.4×10^{-3} (8.0×10^{-4})^b	0.99 (0.99)^b
BTO_2	0.40 (0.64)^b	1.9×10^{-3} (1.2×10^{-3})^b	0.97 (0.99)^b
BTO_3	0.30 (0.70)^b	3.3×10^{-3} (1.0×10^{-3})^b	0.91 (0.98)^b

^{a} k – first order rate constant calculated from $\ln(C/C_0)=-kt$, where C_0 and C (mg L^{-1}) are the concentrations of RhB at time t (min); ^{b} values in parenthesis for 2nd photoreaction cycle

Figure S1. Elemental area-mappings for BTO, BTO_1 and BTO_3, representative examples

BTO

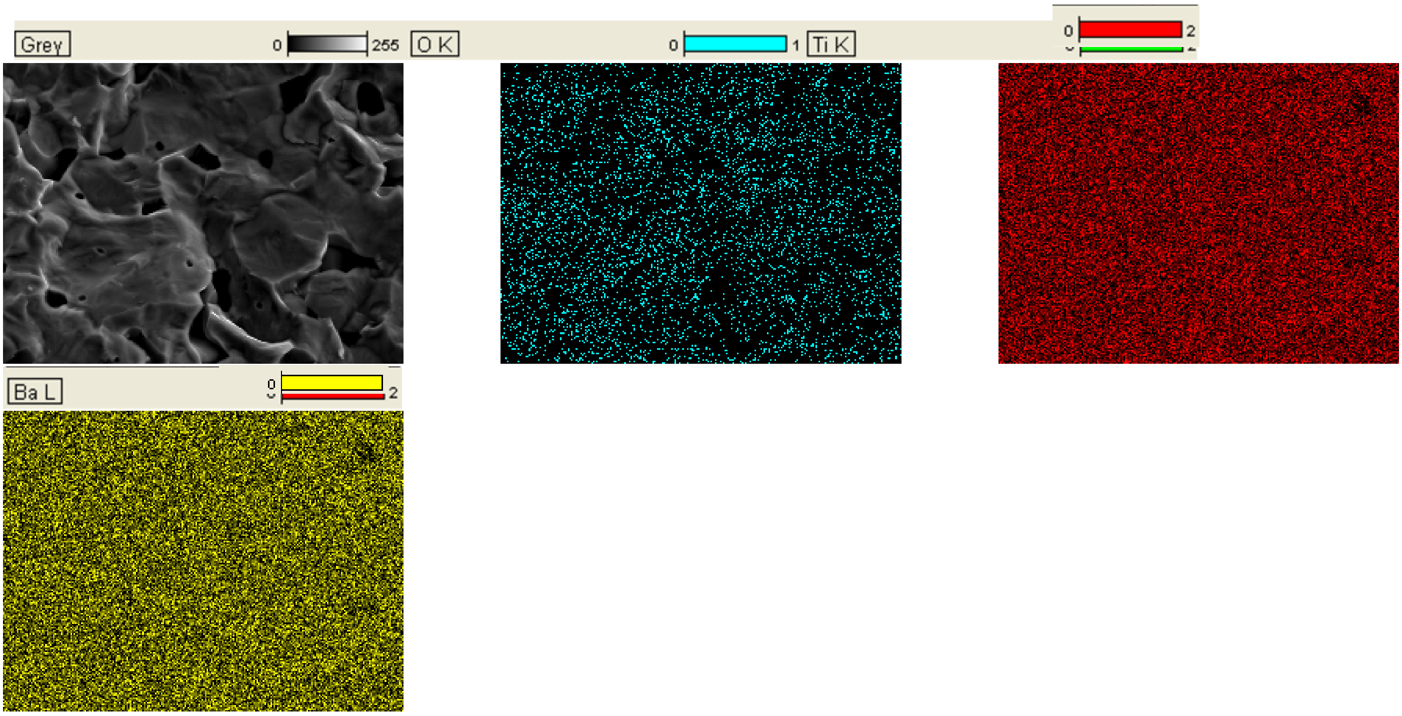

BTO_1

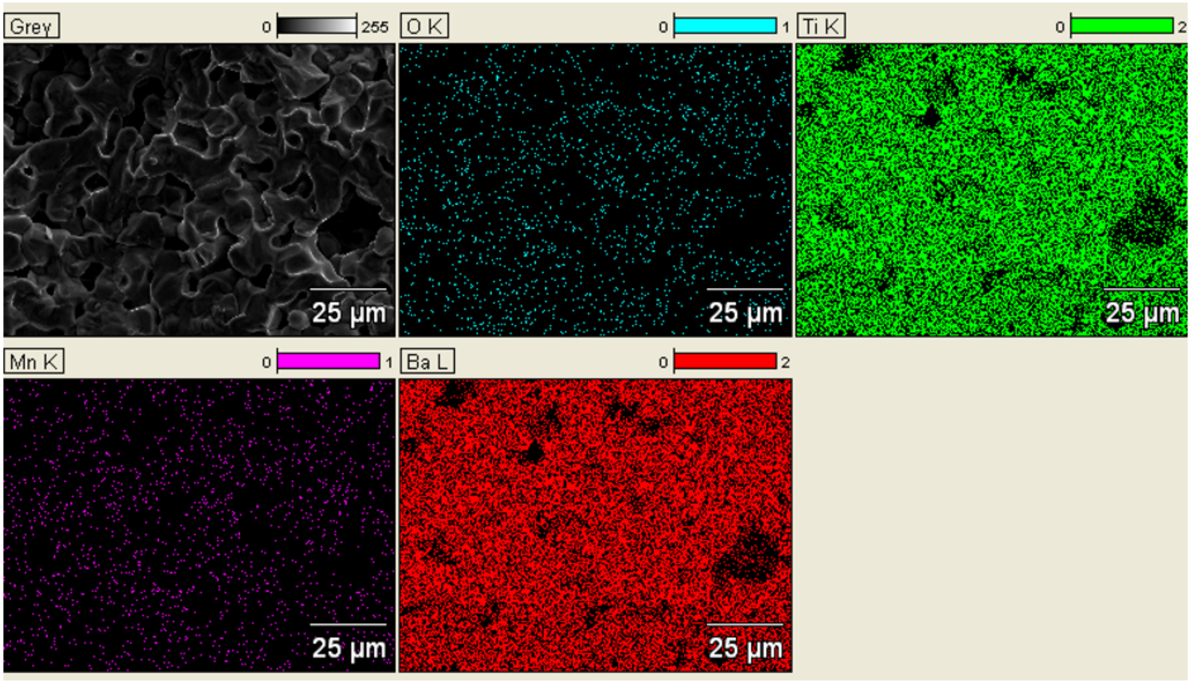

BTO_3

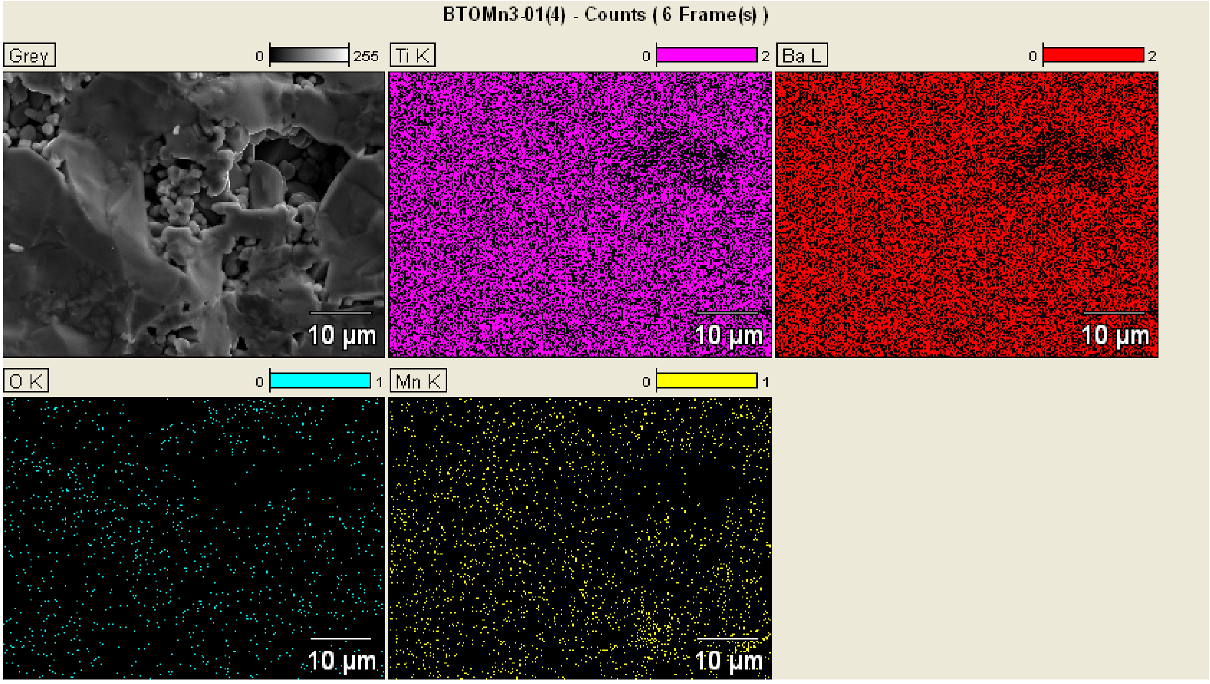

Figure S2. UV-Vis absorption spectra of $BaTiO_3$ ceramics: BTO (a), BTO_1 (b), BTO_2 (c) and BTO_3 (d) in 220-500 cm^{-1} range in Nujol mul

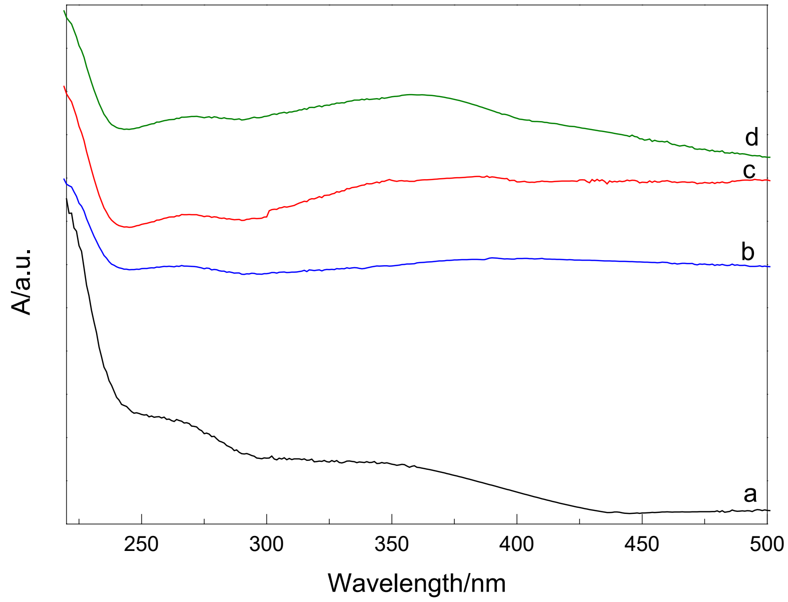

Figure S3. UV-Vis absorption spectra of RhB dye solution with different irradiation time using BTO_1 as photocatalyst

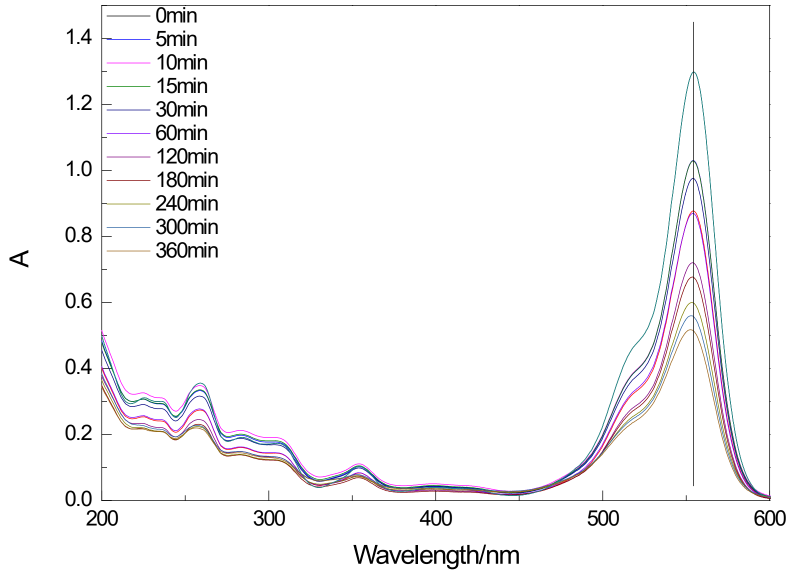

Figure S4. UV-Vis absorption spectra of RhB dye solution with different irradiation time using BTO_2 as photocatalyst

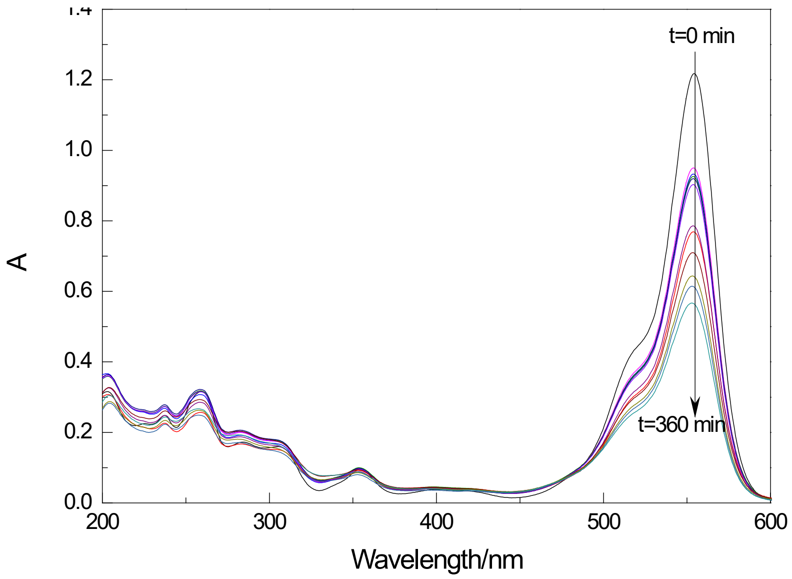

Figure S5. UV-Vis absorption spectra of RhB dye solution with different irradiation time using BTO_3 as photocatalyst

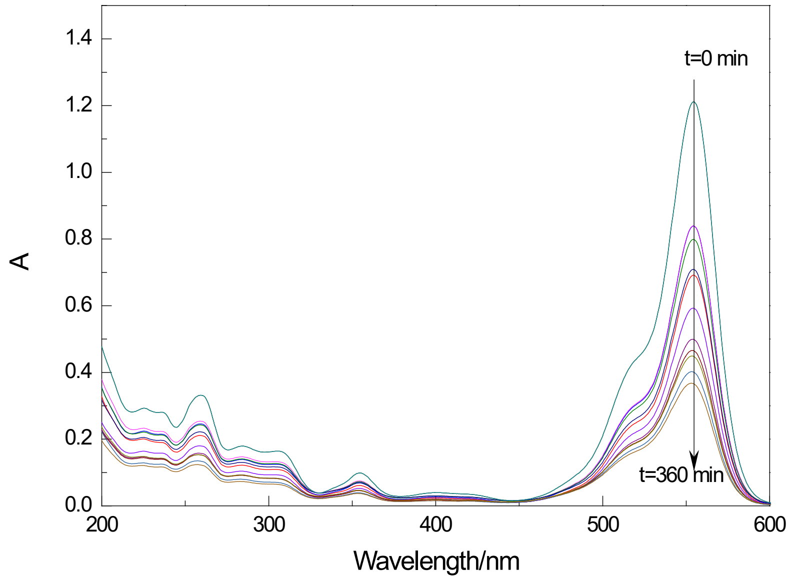

Figure S6. FTIR spectra of BTO_1 (a), BTO_2 (b), BTO_3 (c) and BTO (d) after second photocatalytic cycle.

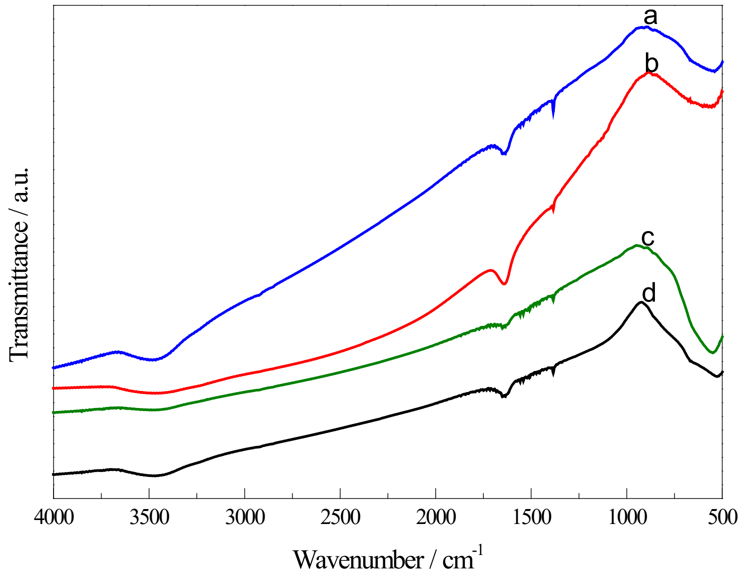

Supplement: Supplementary file 1 [file materials-14-03152-s001.zip › materials-1240771-supplementary.pdf]
